# Can competitive effects and responses of alien and native species predict invasion outcomes?

**DOI:** 10.1016/j.fmre.2024.05.001

**Published:** 2024-05-12

**Authors:** Tingting Wu, Yuanzhi Li, Marc W. Cadotte, Oscar Godoy, Chengjin Chu

**Affiliations:** a State Key Laboratory of Biocontrol, School of Life Sciences, Sun Yat-sen University, Guangzhou 510275, China; b State Key Laboratory of Biocontrol, School of Ecology, Shenzhen Campus of Sun Yat-sen University, Shenzhen 518107, China; c Department of Biological Sciences, University of Toronto-Scarborough, 1265 Military Trail, Toronto, ON M1C 1A4, Canada; d Departamento de Biología, Instituto Universitario de Ciencias del Mar (INMAR), Universidad de Cádiz, E-11510 Puerto Real, Spain

**Keywords:** Competitive effects, Competitive responses, Invasion outcomes, Modern coexistence theory, Plant invasion, Ricker model

## Abstract

The relative competitive ability of native and alien species, which consists of competitive effect (CE) and response (CR), has often been invoked as a key determinant of invasion success. Previous studies have reported that an alien species with a high CE and/or a low CR would successfully invade a native species. However, no studies have yet empirically examined the hypothesis or tested the consistency of invasion outcomes predicted by the CE-CR framework and modern species coexistence theory (MCT). To fill this research gap, we conducted a pairwise competition experiment between five alien and five native species, quantified CE and CR based on their biomass in the absence and presence of *one* competitor, and predicted invasion outcomes based on both CE-CR and MCT frameworks. We have demonstrated theoretically that the CE and CR frequently measured in previous work are only approximations of interspecific competitive coefficients, and thus could not completely predict the invasion outcomes. As we expected, the invasion outcomes predicted by the CE-CR framework were partially consistent with the predictions by the MCT framework. Specifically, aliens with low CR and high CE tended to exclude natives, while aliens with high CR and low CE tended to be excluded by natives according to MCT. In contrast, pairs of stable coexistence and priority effects did not conform to the theoretical expectation. Despite the theoretical defects of the CE-CR framework, it can provide some useful value in predicting the invasion outcomes, especially when intrinsic growth rate and intraspecific competition coefficients are not available. Our study is the first to compare invasion outcomes separately derived from qualitative (the CE-CR framework) and quantitative (the MCT framework) methods. We recommend that future research should adopt quantitative approaches such as MCT as far as possible, to more comprehensively understand and predict the biotic outcomes of interacting species.

## Graphical abstract

**Figure:**
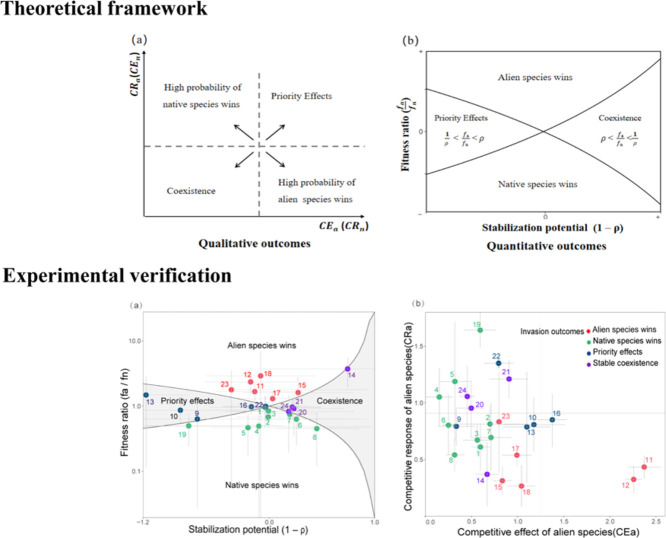

## 1 Introduction

Plant invasion is one of the major threats to the world’s biodiversity, economy, and social development, and has attracted great research attention [[1], [2], [3], [4]]. Various hypotheses have been proposed to explain the success of plant invasions [[5], [6], [7]]. The novel weapons hypothesis claims that alien species can secrete specific allelopathic chemicals that suppress native species, which increases their competitive advantages [8,9]. Furthermore, the enemy release hypothesis predicts that when an alien species is introduced to a new environment, it grows in number and expands in space due to lack of other natural predators, enhancing the fitness advantage of the alien species [[10], [11], [12]]. The evolution of increased competitive ability hypothesis suggests that alien species that arrive in a new environment will transfer the materials and energy used for defense to growth and reproduction, thus enhancing their competitive ability [[13], [14], [15]]. All of the above hypotheses highlight that the competitive ability of alien species relative to native species is a key mechanism for the success of invasions.

**Fig. 1:**
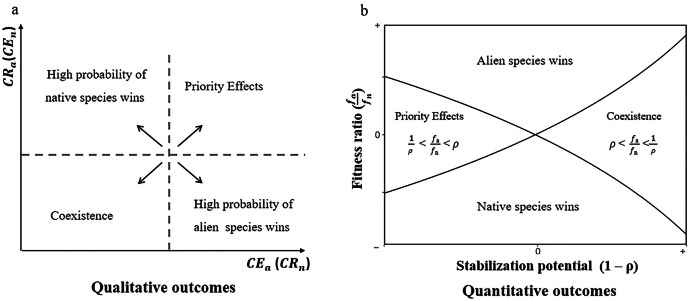
**The conceptual diagram to predict the invasion outcomes of the CE-CR framework (a) and the MCT framework (b).** Panel (a) qualitatively shows the invasion outcomes based on the competitive effects and responses between alien and native species; ${\text{CE}}_{a}$ represents the competitive effect of alien species on native species (i.e., the competitive response of native species to alien species; ${\text{CR}}_{n}$); ${\text{CR}}_{a}$ represents the competitive response of alien species to native species (i.e., the competitive effect of native species on alien species; ${\text{CE}}_{n}$). Arrows illustrate the possibility of invasion outcomes of competitive effect and response of alien species to native species. Panel (b) quantitatively shows the invasion outcomes based on the modern species coexistence theory. The x-axis represents the stabilization potential (1−ρ) and the y-axis represents the fitness ratio ($f_{a}$/$f_{n}$); the solid lines represent the boundary where ($f_{a}$/$f_{n}$) equals ρ and 1/ρ, respectively. The upper and lower white areas indicate the regions where parameter combinations result in the dominance of alien and native species, respectively; the right and left areas indicate regions of stable coexistence and priority effects, respectively.

The relative competitive ability of an alien to a native could be divided into two components: the ability of the aliens to suppress the growth of natives (competitive effect, CE), and ability of the aliens to tolerate competition from the native species without a reduction in growth (competitive response, CR). Tolerance measures the ability of aliens not to reduce their growth in the presence of native species. Many studies have explored how the CR and CE values of the aliens are related to their functional traits [[16], [17], [18], [19], [20], [21]], and whether CE and CR values change with invasion stages and residence time [22,23]. Importantly, because the CE and CR framework describes the ability of a species to grow in the presence of *one* competitor, some studies claim that it can be used to predict competitive outcomes between an alien and a native species [[24], [25], [26]] (Fig. 1a). These outcomes depend on the relative value of CE and CR between an alien and a native species. Specifically, when an alien has a low CR value (not sensitive to a native), it should successfully invade a native species (bottom of Fig. 1a). After invasion, the alien species with a high CE value should competitively exclude the native (bottom right of Fig. 1a), while the alien species with a low CE value will stably coexist with the native (bottom left of Fig. 1a). In contrast, the alien species with a high CR value (sensitive to natives) cannot invade native species (top of Fig. 1a). In this case, the native will inevitably exclude the alien with low CE value (top left of Fig. 1a), while their competitive outcome depends on their initial abundance (We refer this to priority effect as demonstrated [27], top right of Fig. 1a) when the alien has high CE value. Surprisingly, no empirical study has explicitly examined the above CE-CR framework, probably because it does not specify the exact thresholds of CE (whether the alien excludes the native or not) and CR (whether the alien could invade the native or not) to accurately predict invasion outcomes.

The CE-CR framework assumes that alien and native species compete for the same limited resources or space, or be suppressed by the same natural enemies. Therefore, the species with a higher ability to grow and suppress the other is the superior competitor. However, species can also differ in many ways, such as the resources they use, their natural enemies, or the timing of their biological events [28,29]. These differences can promote niche differences that stabilize the dynamics of competitors and promote their coexistence. According to modern coexistence theory (hereafter MCT, [30,31]), it is well known that the invasion outcomes between an alien and a native species could be determined by their niche difference and fitness difference (Fig. 1b) [32,33]. In the absence of niche differences, fitness differences determine the superior competitor, such as in the case of the CE-CR framework. Specifically, MCT posits that coexistence between the alien and the native occurs only when their niche difference exceeds the fitness difference. The alien always wins over the native when its fitness advantage overcomes their niche difference. Therefore, the alien can successfully invade the native, either because they have large niche differences or because the alien has a much greater fitness than the native species. Otherwise, the alien cannot invade the native. However, the impact of aliens on natives will differ. While the invasion by aliens with large niche differences will have little impact on natives, the invasion associated with large fitness differences will tend to reduce growth and population of natives, potentially excluding natives from the community.

Although MCT provides a wide range of predictions for competitive outcomes between aliens and natives, the information required to make these predictions is exhaustive. To calculate niche and fitness differences between species, we need information for the simplest models that describe the population dynamics of interacting species, including at least the intrinsic population growth rate for each species ($\lambda_{a}$ and $\lambda_{n}$), as well as their intraspecific ($\alpha_{aa}$ and $\alpha_{nn}$) and interspecific ($\alpha_{an}$ and $\alpha_{na}$) competitive coefficients (Box 1). Instead, as we show in Box 1, the CE and CR of alien species are just approximations of interspecific competitive coefficients when not considering the density of neighboring species, hence it is self-evident that the CE-CR framework cannot completely predict invasion outcomes (competitive outcomes between a native species and an alien species) according to MCT. Despite the weakness of the CE-CR framework, it still provides some insight into invasion outcomes (Fig. 1a) and might be useful in practice for its simplicity, especially when we do not have information on intrinsic growth rates and/or intraspecific competitive coefficients.

Whether the CE-CR framework can be used to accurately predict competitive outcomes in practice, i.e., whether it aligns with theoretical predictions of the MCT framework (Box 1) remains largely unexplored. To this end, we conducted pairwise competition experiments between five alien and five native species. First, we calculated the CE and CR values of each pair of native-alien species and qualitatively predicted their invasion outcomes according to the CE-CR framework (Fig. 1a). Second, we estimated the interaction coefficients of each pair of native-alien species according to the experimental data and quantitatively predicted their invasion outcomes based on the MCT framework (Fig. 1b). Specifically, we aim to address the following questions: (1) How do CE and CR affect the invasion outcomes between an alien species and a native species? (2) Whether the invasion outcomes predicted based on the CE and CR values were approximately consistent with those predicted by the modern species coexistence theory?

Box 1 **The CE and CR values frequently measured in previous studies are only approximations of interspecific competitive coefficients.**

**Fig. 2:**
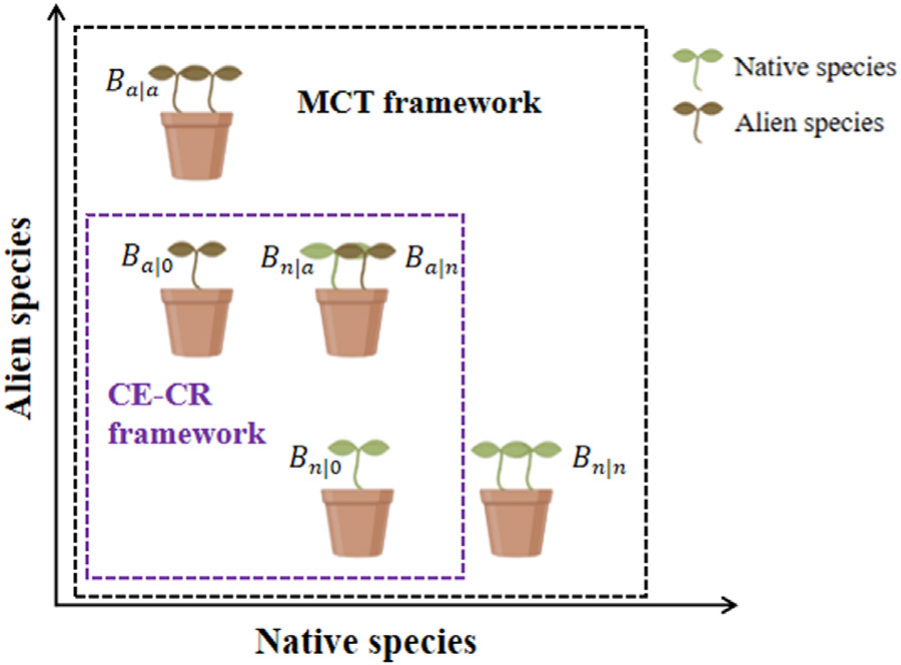

**Fig. 2. Comparison of experimental designs for CE-CR and MCT frameworks.** Under the CE-CR framework, each native (alien) species must be grown alone and with *one* heterospecific alien (native) competitor to calculate $B_{n|0}$ ($B_{a|0}$) and $B_{n|a}$ ($B_{a|n}$), respectively. While in the MCT framework, each native (alien) species must be grown alone to estimate intrinsic growth rate ($\lambda_{n}$/$\lambda_{a}$), and with at least one conspecific and heterospecific competitor to estimate$\;\text{intraspecific}\;(\alpha_{nn}/\alpha_{aa}$) and interspecific coefficients ($\alpha_{na}/\alpha_{an}$), respectively.

According to previous studies, the competitive effect (CE) and competitive response (CR) of a given pair of native-alien species were calculated based on an experiment of planting one native individual alone, one alien individual alone, and one native individual and one alien together (Fig. 2, N, A, NA). $B_{n|0}$ ($B_{a|0}$) is the biomass of one native (alien) individual grown alone, and $B_{n|a}$ ($B_{a|n}$) is the biomass of one native (alien) individual in the presence of one alien (native) individual. Assuming the same initial biomass, the ${\text{CE}}_{a}\;$ (CE of the alien species) equals the ${\text{CR}}_{n}$ (CR of the native species) and the ${\text{CR}}_{a}\;$ (CR of the alien species) equals the ${\text{CE}}_{n}$ (CE of the native species) are quantified as:

(1) $$\left\{ \begin{array}{c} {\text{CE}}_{a}={\text{CR}}_{n}=\ln\left(\frac{B_{n|0}}{B_{n|a}}\right)\\ {\text{CR}}_{a}={\text{CE}}_{n}=\ln\left(\frac{B_{a|0}}{B_{a|n}}\right) \end{array}\right.$$

In addition to the three planting combinations (N, A, NA) used to estimate CE and CR values, planting two individuals of a native species (NN) and two individuals of an alien species (AA) is required to estimate competitive coefficients between the native and the alien species in a population dynamical model, e.g., the Ricker competition model [39]:

(2) $$\left\{ \begin{array}{c} \text{ln}\;\left(B_{a}\right)=\ln\left(\lambda_{a}\right)-\alpha_{aa}N_{a}-\alpha_{an}N_{n}\\ \text{ln}\;\left(B_{n}\right)=\ln\left(\lambda_{n}\right)-\alpha_{na}N_{a}-\alpha_{nn}N_{n} \end{array}\right.$$

$B_{a}$ ($B_{n}$) is the biomass of an alien (a native) individual grown alone or in the presence of *one* competitor, $\lambda_{a}$ ($\lambda_{n}$) is the potential biomass growth of the alien (native) species grown alone,$\;\alpha_{aa}$, $\alpha_{an}$, $\alpha_{na}$ and $\alpha_{nn}$ are the intraspecific and interspecific competitive coefficients between the native and alien species, $N_{a}$ and $N_{n}$ (either 0 or 1 when estimating CE and CR) are the neighbor densities of the alien and native species. Apply the Ricker competition model to estimate CE and CR, and we get:

(3) $$\left\{ \begin{array}{c} \ln\left(B_{a|0}\right)=\ln\lambda_{a}\;\\ \ln\left(B_{a|n}\right)=\ln\lambda_{a}-\alpha_{an} \end{array}\right.\text{and}\;\left\{ \begin{array}{c} \ln\left(B_{n|0}\right)=\ln\lambda_{n}\;\\ \ln\left(B_{n|a}\right)=\ln\lambda_{n}-\alpha_{na} \end{array}\right.$$

and we can then derive the following equations:

(4) $$\left\{ \begin{array}{c} \alpha_{an}=\ln\left(\frac{B_{a|0}}{B_{a|n}}\right)={\text{CR}}_{a}\\ \alpha_{na}=\ln\left(\frac{B_{n|0}}{B_{n|a}}\right)={\text{CE}}_{a} \end{array}\right.$$

Therefore, the CE and CR values are just approximations of interspecific competitive coefficients between a native and an alien ($\alpha_{na}\;and\;\alpha_{an}$), which are estimated from the modified Ricker competition model when a native (or an alien) grows in the absence of a neighbor and in the presence of *one* heterospecific neighbor.

Under the MCT framework, the competitive outcomes can be predicted based on the niche difference and the fitness difference between the alien and native species. The niche difference is calculated as 1−ρ, where ρ (niche overlap) was quantified as [34]:

(5) $$\rho =\sqrt{\frac{\alpha_{na}\alpha_{an}}{\alpha_{aa}\alpha_{nn}}}$$

Similarly, their fitness difference is calculated as [35]:

(6) $$\frac{f_{a}}{f_{n}}=\frac{\ln\lambda_{a}}{\ln\lambda_{n}}\sqrt{\frac{\alpha_{na}\alpha_{nn}}{\alpha_{aa}\alpha_{an}}}$$

The fitness difference is composed of two components: the demographic differences ($\frac{\ln\lambda_{a}}{\ln\lambda_{n}}$) describe whether the intrinsic growth rate of the alien is higher than that of the native, and the competitive response differences ($\sqrt{\frac{\alpha_{na}\alpha_{nn}}{\alpha_{aa}\alpha_{an}}}$) indicate, on average, whether aliens can better tolerate competition than natives. Therefore, an alien species can exhibit higher fitness compared to a native species because it either has a high intrinsic growth rate, a low response to reduced growth in the presence of a native competition, or a combination of both. The stable coexistence of a native-alien pair is expected when $\rho \;<\;\frac{\kappa_{a}}{\kappa_{n}}\;<\;\frac{1}{\rho }$, whereas the priority effect occurs when $\rho \;>\;\frac{\kappa_{a}}{\kappa_{n}}\;>\;\frac{1}{\rho }$ (Fig. 1b). Therefore, it is theoretically obvious that the CE and CR values (interspecific competitive coefficients) alone cannot accurately predict the invasion outcome.

Alt-text: Unlabelled box

## 2 Materials and methods

### 2.1 Experimental design and measurements

Our experiment was carried out in a greenhouse in the Chebaling Nature Reserve (114°09′04″−114°16′46″ E, 24°40′29″−24°46′21″ N) in Southern China. This area belongs to the subtropical monsoon climate, with an average annual temperature of 19.6 °C and annual precipitation of 1,468 mm. The experiment consisted of pairwise competitions between five native species and five alien species that are common in this region according to the Chinese list of invasive species (Table 1). These native and alien species frequently co-occur in the field, exhibiting broad adaptability and requiring no specific environmental or nutrient conditions. The five alien species are all naturalized in China, but here we refer to them collectively as “alien species” due to their varying levels of establishment success [36]. Among them, three species, i.e., *Bidens pilosa, Ageratum conyzoides, Praxelis clematidea* are recognized as invasive species in China, while the other two are naturalized species [37]. In March 2021, the seeds of these species were collected in the field and then sown in seedling trays (60 cm × 30 cm × 10 cm) filled with a sterilized mixture of sand and vermiculite (1:1). After one month, for each pair of native-alien species (25 pairs in total), seedlings of native and alien species with the same initial plant height (around 2 cm) were transplanted into pots (14.5 cm × 18.5 cm × 20.5 cm) filled with well-mixed, sieved field soil, according to the minimum competition experiment design [35] (Box 1, Fig. 2). Seedlings were planted with combinations of N (one seedling of native species), NN (two seedlings of same native species), A (one seedling of alien species), AA (two seedlings of the same alien species) and AN (one seedling of native species and one seedling of alien species). We set six replicates for each combination. For each replicate, species and its corresponding species pair were treated as a block. Thus, there were six blocks in the experiment, with 45 pots in each block, resulting in 270 pots together.

**Table 1:** **The species involved in the study**.

Species	Abbreviation	Family	Status	Perenniality
*Emilia sonchifolia* (L.) DC	Emi.son	Asteraceae	Native	annual
*Eleusine indica (*L.*)* Gaertn	Ele.ind	Amaranthaceae	Native	annual
*Lophatherum gracile*	Lop.gra	Gramineae	Native	annual
*Achyranthes bidentata* Blume	Ach.bid	Amaranthaceae	Native	perennial
*Anisomeles indica* (L.) Kuntze	Ani.ind	Labiatae	Native	annual
*Bidens pilosa* L.	Bid.pil	Asteraceae	Alien	annual
*Ageratum conyzoides* L	Age.con	Asteraceae	Alien	annual
*Praxelis clematidea* (Griseb.) R. M. King et H. Rob	Pra.cle	Asteraceae	Alien	annual
*Solanum photeinocarpum* Nakamura et S. Odashima	Sol.pho	Solanaceae	Alien	annual
*Amaranthus viridis* L.	Ama.vir	Amaranthaceae	Alien	annual

Within the week after transplantation, we replaced dead seedlings with new individuals. All pots were placed uniformly with 15 cm between them and were regularly watered and weeded every two days for the first two weeks and watered as needed during the experiment. The positions of the pots within each replicate were randomly changed every two months to maximize the consistency of the external environmental conditions. After five months of growth, we harvested the aboveground biomass of all individual plants and dried them at 60 °C for three days to a constant weight, as the underground roots of plants were intertwined and difficult if not impossible to assign to each individual.

### 2.2 Invasion outcomes based on the CE–CR framework

We calculated the CE and CR value for each pair of native-alien species according to biomass production in the absence or presence of a competitor within each block (Eq. 1 in Box 1) [26,38]. Therefore, all alien species have six replicate CE and CR values when grown with native species. Then, the CE and CR values were averaged among the six replicates for each pair of native-alien species. The native-alien invasion outcomes could then be qualitatively predicted based on the CE-CR framework (Fig. 1a).

### 2.3 Invasion outcomes based on the MCT framework

In addition to obtaining qualitative invasion outcomes based on the CE-CR framework, we quantitatively predicted invasion outcomes between an alien and a native species under the MCT framework (Fig. 1b). For each pair of native-alien species, we estimated the interaction coefficients and intrinsic growth rates based on their biomass productions in the absence or presence of *one* competitor, using the Ricker competition model in linearized version [39], which has been widely used to model plant competitive population dynamics [40]:

(7) $$\ln\left(\frac{B_{ai,t}}{B_{ai,0}}\right)=\ln\left(\lambda_{ai}\right)-\alpha_{ai,ai}N_{ai}-\sum_{i,j=1}^{5}\left(\alpha_{ai,nj}N_{nj}\right)$$

$B_{ai,0}$ and $B_{ai,t}$ are the aboveground biomass of an individual of the alien species *i* at the beginning and end of the experiment, respectively; $\ln(\lambda_{ai})\;$is the potential biomass growth of the alien species *i* in the absence of neighbors; $\alpha_{ai,nj}$ is per capita effect of the native species *j* on the alien species *i*; $N_{nj}$ (either 0 or 1 in our experiment) is the density of the native species *j*. Similarly, the potential biomass growth of native species $\ln(\lambda_{nj})\;$and the per capita effect of the alien species *i* on native species *j* ($\alpha_{nj,ai}$) could also be estimated from Eq.7 when an individual of the native species was treated as the focal species. The niche differences and fitness differences were calculated for all native-alien pairs according to the Eqs. 5 and 6 in Box 1, except for one pair (Ele. ind-Ama. vir) in which interactions were positive and niche differences could not be calculated due to the square root term. In addition, we provided error bars for niche and fitness differences calculated by propagating the standard error of each parameter of the annual plant model that was obtained from the conditions of the pot experiment or the maximum likelihood estimations [41]. All analyses were performed in *R*. 4.0.3 [42].

## 3 Results

**Fig. 3:**
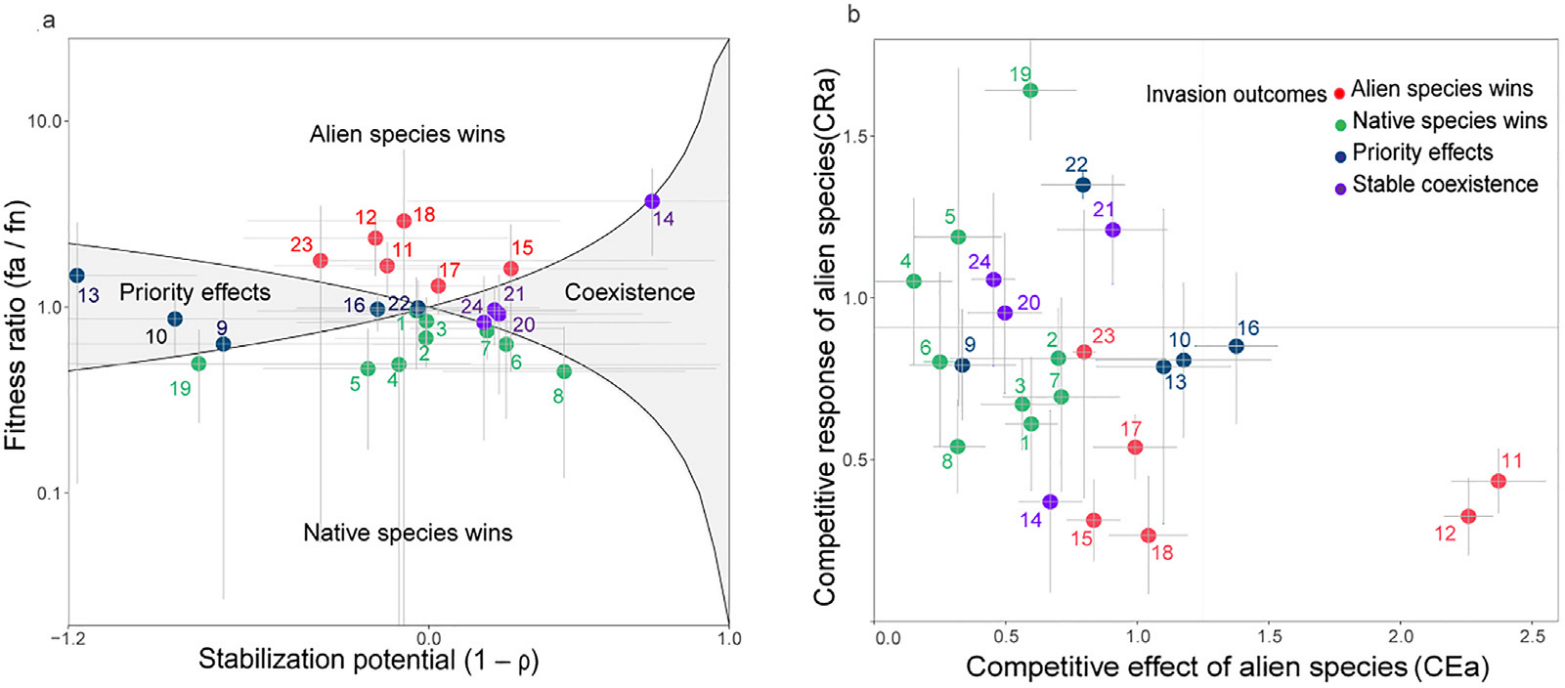
**Quantitative and qualitative invasion outcomes for each pair of native and alien species (denoted by a single point) based on the MCT framework (a) and CE-CR framework (b), respectively.** Panel (a) the x-axis represents the stabilization potential (1−ρ) and the y-axis represents the fitness ratio ($f_{a}$/$f_{n}$); the solid lines represent the boundary where ($f_{a}$/$f_{n}$) equals ρ and 1/ρ, respectively. Error bars were calculated from propagating the standard error of each parameter of the annual plant model. Panel (b) the x-axis represents the competitive effect of alien species on native species (${\mathrm{CE}}_{a}$), and the y-axis represents the competitive response of alien species to native species (${\mathrm{CR}}_{a}$). Error bars represent 95% confidence intervals. In the two figures, red and green colors represent alien and native species wins, respectively; purple and dark blue represent stable coexistence and priority effects, respectively. The numbers 1–24 correspond to the following species pairs: 1 Emi.son_Bid.pil; 2 Emi.son_Age.con; 3 Emi.son_Pra.cle; 4 Emi.son_Sol.pho; 5 Emi.son_Ama.vir; 6 Ele.ind _Bid.pil; 7 Ele.ind _Age.con;8 Ele.ind _Pra.cle; 9 Ele.ind _Sol.pho; 10 Lop.gra_Bid.pil; 11 Lop.gra_Age.con; 12 Lop.gra_Pra.cle; 13 Lop.gra_Sol.pho; 14 Lop.gra_Ama.vir; 15 Ach.bid_Bid.pil; 16 Ach.bid_Age.con; 17 Ach.bid_Pra.cle; 18 Ach.bid_Sol.pho; 19 Ach.bid_Ama.vir; 20 Ani.ind_Bid.pil; 21 Ani.ind_Age.con; 22 Ani.ind_Pra.cle; 23 Ani.ind_Sol.pho; 24 Ani.ind_Ama.vir.

Based on the MCT framework, we predicted stable coexistence for four pairs, competitive exclusion for 15 pairs (aliens win in six pairs and natives win in nine pairs), and priority effect for five pairs (Fig. 3a). The aliens could invade the natives in ten out of 24 pairs (aliens win in six pairs, and aliens in four pairs coexist with natives) (Fig. 3a). The invasion outcomes qualitatively predicted by the CE-CR framework were partially consistent with the quantitative outcomes predicted by the MCT framework (Fig. 3b). As expected, the aliens with low CR and high CE (bottom right of Fig. 3b) tended to exclude natives, while aliens with high CR and low CE (top left of Fig. 3b) tended to lose to natives. In contrast, pairs of stable coexistence and priority effects were not located in the expected bottom left and top right regions under the CE-CR framework (Fig. 3b).

**Fig. 4:**
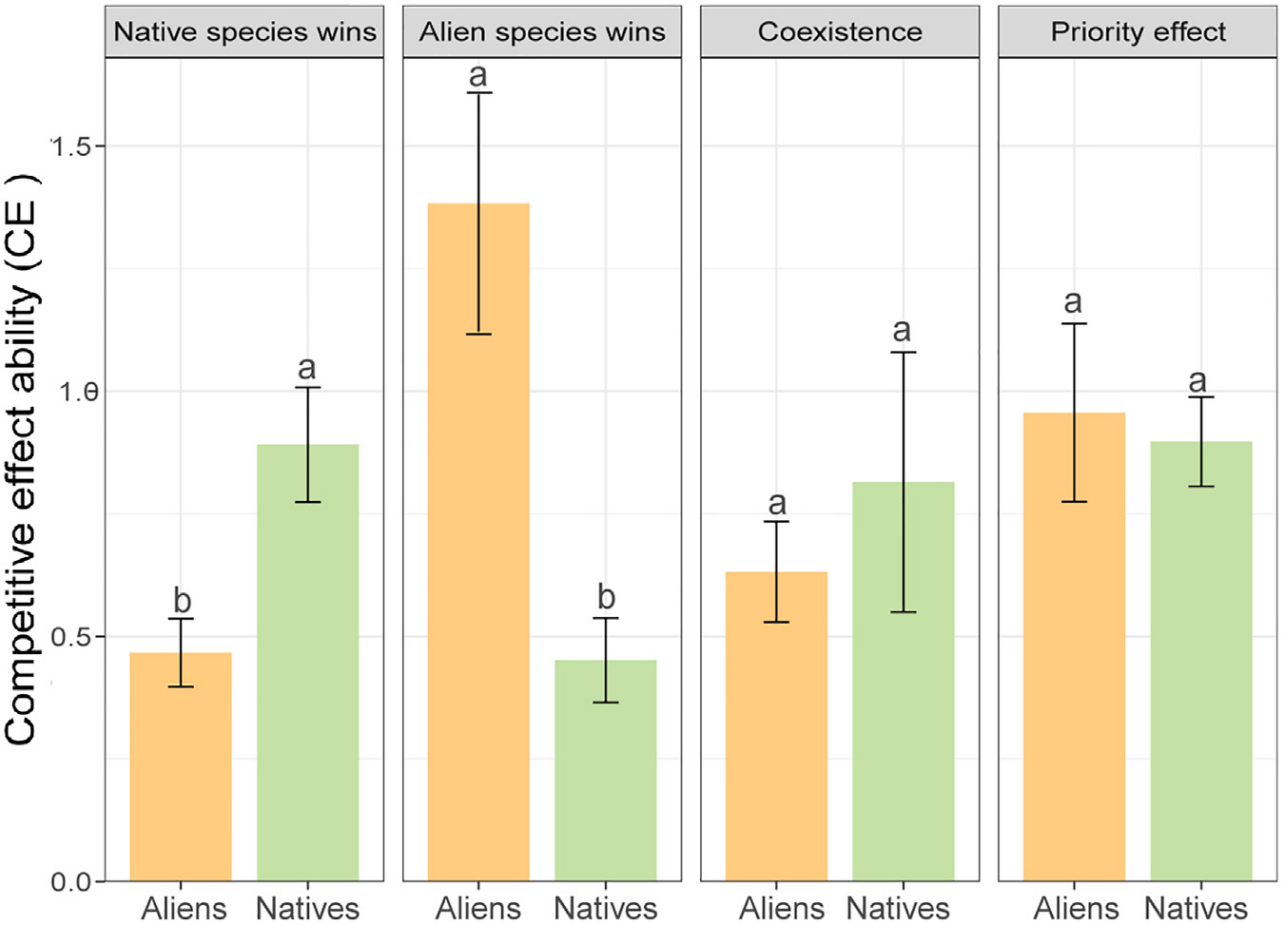
**The competitive effects ability of alien and native species for all pairs of species under different invasion outcomes based on the MCT framework.** Orange and light green represent the competitive effect ability of alien and native species, respectively, under different competitive outcomes. Bars indicate mean ± SE. The T test was used to assess statistical differences between group means and different lowercase letters indicate significant differences (*p* < 0.05).

According to the invasion outcomes under the two frameworks, we found that the CE value of the aliens was higher than that of the natives when the aliens won over the natives, and vice versa (Fig. 4, *p* < 0.05). Additionally, no significant differences in CE between aliens and natives were observed when stable coexistence and the priority effect occurred (Fig. 4, *p* > 0.05). A similar pattern could be derived when comparing the CR value between aliens and natives because the CE of an alien is equal to the CR of a native, and the CE of a native is equal to the CR of an alien species (Eq. 1).

## 4 Discussion

Our study explicitly explores, for the first time, whether the prediction of the invasion outcomes based on the CE (competitive effect)-CR (competitive response) framework is consistent with the MCT framework. As we theoretically demonstrated in Box 1, CE and CR measured based on biomass in the absence and presence of *one* competitor are only approximations of interspecific competition and consequently, cannot completely predict competitive outcomes between an alien and a single native species due to the inability to account for intraspecific effects. This theoretical derivation was empirically validated by comparing the invasion outcomes predicted by the CE-CR and MCT frameworks (Fig. 3). In other words, although the CE-CR framework cannot fully predict the invasion outcomes in theory, it can provide some useful value in predicting the invasion outcomes to some extent in practical situations.

Specifically, alien species were predicted to competitively exclude native species in six out of 24 pairs under the MCT framework (Fig. 3a), where the alien species have higher CE or lower CR relative to the native species (Figs. 3b, 4). In contrast, the alien species were predicted to be competitively excluded by the native species in nine out of 24 pairs (Fig. 3a), where the alien species tend to have lower CE or higher CR relative to the native species (Figs. 3b, 4). This indicates that these two frameworks are roughly consistent in predicting invasion outcomes based on species’ competitive abilities. However, the CE-CR framework only highlights interspecific competitive ability while neglecting the significant role of intraspecific competitive ability in predicting invasion outcomes. Furthermore, this result is consistent with previous studies that showed that successfully invading alien species should be more competitive (with high CE and/or low CR) than natives [16,43,44], and implies that the competitive ability (CE and CR values) is a key determinant of invasion outcomes [[45], [46], [47], [48]]. Notably, stable coexistence and priority effect occur for four and five species pairs, respectively (Fig. 3a), for alien and native species who are equally competitive (Fig. 4). In this scenario, the competitive outcomes of these pairs depended on the degree of niche overlap (ρ) [27]. When ρ was between 0 and 1, the alien and native species would coexist stably. When ρwas greater than 1, the invasion outcomes of alien and native species depended on their initial abundance. Therefore, the competitive ability between alien and native species is a potentially critical but not the only factor that affects and predicts invasion outcomes. Other factors, such as niche difference, could be a crucial factor in predicting invasion outcomes and deserve further exploration [[49], [50], [51], [52], [53], [54]].

The discrepancy between CE-CR and MCT frameworks in predicting invasion outcomes does not dismiss the potential value of the CE-CR framework. The CE-CR framework predicted that the alien species with high CE and low CR tended to exclude the natives, while the aliens with high CR and low CE tended to be excluded by the native species, and they were both distributed in the exclusion regions in the MCT framework (Fig. 3). This indicated that although the CE-CR framework has theoretical weakness (i.e., CE and CR of species are only proxies for interspecific competition), it can be used to roughly predict invasion outcomes to some extent. After all, this framework requires significantly less information, especially in simple interspecific competition experiments or the competition between two single individuals of different species without considering neighbor density. Many studies showed that CE and CR were the key determinants of invasion outcomes [18,55,56], especially in the absence of additional information such as intrinsic growth rate and intraspecific competition coefficients. However, compared with the invasion outcomes predicted by the MCT framework, pairs of stable coexistence and priority effects were not located in the expected regions, respectively, under the CE-CR framework (Fig. 3b). The possible reason may be attributed to two factors. On the one hand, the CE-CR framework cannot specify the exact thresholds of CE and CR, resulting in a rough and qualitative prediction of invasion outcomes (Fig. 1a). This indicated that other quantitative methods are required if we want to accurately predict the eventual winners and losers of each species pair. On the other hand, this framework emphasizes interspecific competition while neglecting the role of intraspecific competition, as the experimental design for measuring CE and CR in previous studies only involved inter-specific planting (Box 1, Fig. 2). This suggests that the CE-CR framework could be useful when intraspecific competition data is lacking, but that the MCT framework is an improved approach if it can be successfully parameterized.

It is important to mention that, though the species we selected in the present work co-occur in natural communities, only four pairs of alien and native species could stably coexist, as predicted by the MCT framework (Fig. 3a). This inconsistency between observed patterns and predictions from MCT has also been frequently reported in previous studies [34,57]. This could be caused by indirect interactions such as higher-order interactions only occurring in multi-species communities [[58], [60], [59]], or potential effects of spatial or temporal fluctuations on coexistence [61,62], or the experimental venue, i.e., pots provide limited opportunities for species to express their niche differences [63]. Meanwhile, positive interactions among aliens may facilitate co-invasion of multiple alien species (i.e., invasion meltdown), or biotic nonnative resistance hindered their successful invasion, indirectly benefiting native species [[64], [65], [66], [67], [68]]. The potential impacts of these ecological processes on invasion outcomes require further empirical and theoretical explorations in future studies, especially in naturally species-rich communities.

In summary, the present study is the first to compare invasion outcomes separately derived from qualitative (the CE-CR framework) and quantitative (the MCT framework) methods, and showed that the invasion outcomes qualitatively predicted based on the CE-CR framework were partially consistent with those quantitatively predicted based on the MCT framework in the absence of neighbor density. However, although the CE-CR framework can qualitatively predict competitive outcomes to some extent, other quantitative methods, such as MCT are required if we aim to accurately predict the eventual winners and losers of each species pair. Of course, this theoretical framework is limited only to competitive systems, which are not able to deal with the case where facilitation between alien and native species occurs [45]. A recently developed structural approach that has been considering different types of interaction could be employed to completely predict the likelihood of invasion outcomes between alien and native species [69], as well as to explore the resistance of multi-species resident communities to invasion or co-invasion of aliens [33].

## Author contributions

CC and TW designed the research; TW conducted the field work and collected data; TW, YL, and CC analyzed the data; TW, YL, and CC wrote the first draft and revised it, with substantial input from OG and MWC. All authors contributed to the writing and editing of the manuscript.

## Declaration of competing interest

The authors declare that they have no conflicts of interest in this work.

## References

[1] Carboni M., Livingstone S.W., Isaac M.E. (2021). Invasion drives plant diversity loss through competition and ecosystem modification. J. Ecol..

[2] Roiloa S.R., Yu F.-H., Barreiro R. (2020). EDITORIAL: Plant invasions: Mechanisms, impacts and management. Flora.

[3] Livingstone S.W., Isaac M.E., Cadotte M.W. (2020). Invasive dominance and resident diversity: Unpacking the impact of plant invasion on biodiversity and ecosystem function. Ecol. Monogr..

[4] Feng Y.L., Du D., van Kleunen M. (2022). Global change and biological invasions. J. Plant Ecol..

[5] Enders M., Havemann F., Ruland F. (2020). A conceptual map of invasion biology: Integrating hypotheses into a consensus network. Glob. Ecol. Biogeogr..

[6] Chiuffo M.C., Moyano J., Policelli N. (2022). Importance of invasion mechanisms varies with abiotic context and plant invader growth form. J. Ecol..

[7] Cadotte M.W., Potgieter L.J., Wang C.J. (2021). Invasion theory as a management tool for increasing native biodiversity in urban ecosystems. J. Appl. Ecol..

[8] Yuan L., Li J.-M., Yu F.-H. (2021). Allelopathic and competitive interactions between native and alien plants. Biol. Invasions.

[9] Zhang Z., Liu Y., Lin Y. (2021). Effect of allelopathy on plant performance: A meta-analysis. Ecol. Lett..

[10] Brian C.A., Godsoe W.K., Bufford J.L. (2022). Can the enemy release hypothesis explain the success of *Rumex* (Polygonaceae) species in an introduced range?. Biol. Invasions.

[11] Brian J.I., Catford J.A. (2023). A mechanistic framework of enemy release. Ecol. Lett..

[12] Costan K., Okarma H., Chmura D. (2020). Enemy pressure exerted on alien and native plants may differ between montane and lowland regions. Arthropod. Plant Interact..

[13] Hiatt D., Flory S.L. (2020). Populations of a widespread invader and co-occurring native species vary in phenotypic plasticity. New Phytol..

[14] Callaway R.M., Lucero J.E., Hierro J.L. (2022). The EICA is dead? Long live the EICA!. Ecol. Lett..

[15] Parker J.D., Burkepile D.E., Hay M.E. (2006). Opposing effects of native and exotic herbivores on plant invasions. Science.

[16] Guido A., Hoss D., Pillar V.D. (2017). Exploring seed to seed effects for understanding invasive species success. Perspect. Ecol. Conserv..

[17] Guido A., Hoss D., Pillar V.D. (2019). Competitive effects and responses of the invasive grass Eragrostis plana in Río de la Plata grasslands: Effects and responses of Eragrostis plana. Austral. Ecol..

[18] Puritty C., Mayfield M., Azcárate F. (2018). Different traits predict competitive effect versus response by Bromus madritensis in its native and invaded ranges. Biol. Invasions.

[19] Tortorelli C.M., Kerns B.K., Krawchuk M.A. (2022). Community invasion resistance is influenced by interactions between plant traits and site productivity. Ecology.

[20] Palma E., Vesk P.A., White M. (2021). Plant functional traits reflect different dimensions of species invasiveness. Ecology.

[21] Guido S.E., Nuñez M.A. (2016). Invasive non-native plants have a greater effect on neighbouring natives than other non-natives. Nat. Plants.

[22] Sheppard C.S., Brendel M. (2021). Competitive ability of native and alien plants: Effects of residence time and invasion status. NeoBiota.

[23] Chen D., van Kleunen M. (2022). Competitive effects of plant invaders on and their responses to native species assemblages change over time. NeoBiota.

[24] Golivets M., Wallin K.F. (2018). Neighbour tolerance, not suppression, provides competitive advantage to non-native plants. Ecol. Lett..

[25] Aschehoug E.T., Brooker R., Atwater D.Z. (2016). The mechanisms and consequences of interspecific competition among plants. Annu. Rev. Ecol. Evol. Syst..

[26] Miller T.E., Werner P.A. (1987). Competitive effects and responses between plant species in a first-year old-field community. Ecology.

[27] Ke P.-J., Letten A. (2018). Coexistence theory and the frequency-dependence of priority effects. Nat. Ecol. Evol..

[28] Ren J., Chen P., Shen C. (2023). Functional and phylogenetic similarities of co-occurring invaders affect the growth of an invasive forb. J. Plant Ecol..

[29] Wang C., Yu Y., Cheng H. (2021). Which factor contributes most to the invasion resistance of native plant communities under the co-invasion of two invasive plant species?. Sci. Total Environ..

[30] Chesson P. (2000). Mechanisms of maintenance of species diversity. Annu. Rev. Ecol. Evol. Syst..

[31] Chesson P. (2018). Updates on mechanisms of maintenance of species diversity. J. Ecol..

[32] MacDougall A.S., Gilbert B., Levine J.M. (2009). Plant invasions and the niche. J. Ecol..

[33] Godoy O. (2019). Coexistence theory as a tool to understand biological invasions in species interaction networks: Implications for the study of novel ecosystems. Funct. Ecol..

[34] Godoy O., Kraft N.J.B., Levine J.M. (2014). Phylogenetic relatedness and the determinants of competitive outcomes. Ecol. Lett..

[35] Hart S.P., Freckleton R.P., Levine J.M. (2018). How to quantify competitive ability. J. Ecol..

[36] Blackburn T.M., Pyšek P., Bacher S. (2011). A proposed unified framework for biological invasions. Trends Ecol. Evol..

[37] Hao Q., Ma S. (2022). Invasive alien plants in China: An update. Plant Divers.

[38] Wang P., Stieglitz T., Zhou D. (2010). Are competitive effect and response two sides of the same coin, or fundamentally different?. Funct. Ecol..

[39] Ricker W.E., Recruitment Stock (1954). Stock and Recruitment.

[40] Zhang Z., van Kleunen M. (2019). Common alien plants are more competitive than rare natives but not than common natives. Ecol. Lett..

[41] Matías L., Godoy O., Gómez-Aparicio L. (2018). An experimental extreme drought reduces the likelihood of species to coexist despite increasing intransitivity in competitive networks. J. Ecol..

[42] Team R.C. (2021). R: A language and environment for statistical computing. MSOR Connect..

[43] Oduor A.M.O. (2022). Native plant species show evolutionary responses to invasion by *Parthenium hysterophorus* in an African savanna. New Phytol..

[44] Gruntman M., Pehl A.K., Joshi S. (2014). Competitive dominance of the invasive plant Impatiens glandulifera: Using competitive effect and response with a vigorous neighbour. Biol. Invasions.

[45] Sheppard C.S. (2019). Relative performance of co-occurring alien plant invaders depends on traits related to competitive ability more than niche differences. Biol. Invasions.

[46] Schultheis E.H., MacGuigan D.J. (2018). Competitive ability, not tolerance, may explain success of invasive plants over natives. Biol. Invasions.

[47] Mesa J.M., Dlugosch K.M. (2020). The evolution of invasiveness: A mechanistic view of trade-offs involving defenses. Am. J. Bot..

[48] Bottollier-Curtet M., Planty-Tabacchi A.-M., Tabacchi E. (2013). Competition between young exotic invasive and native dominant plant species: Implications for invasions within riparian areas. J. Veg. Sci..

[49] Sheppard C.S., Burns B.R. (2014). Effects of interspecific alien versus intraspecific native competition on growth of native woody plants. Plant Ecol..

[50] Farrer E.C., Goldberg D.E. (2011). Patterns and mechanisms of conspecific and heterospecific interactions in a dry perennial grassland. J. Ecol..

[51] Huang F., Lankau R., Peng S. (2018). Coexistence via coevolution driven by reduced allelochemical effects and increased tolerance to competition between invasive and native plants. New Phytol..

[52] Čuda J., Skálová H., Janovský Z. (2015). Competition among native and invasive Impatiens species: The roles of environmental factors, population density and life stage. AoB Plants.

[53] Helsen K., Van Cleemput E., Bassi L. (2020). Inter- and intraspecific trait variation shape multidimensional trait overlap between two plant invaders and the invaded communities. Oikos.

[54] Lee S., Woo S., Kim E. (2020). Differential effect of inter- and intraspecific competition on the performance of invasive and native Taraxacum species. Plant Species Biol..

[55] Besaw L.M., Thelen G.C., Sutherland S. (2011). Disturbance, resource pulses and invasion: Short-term shifts in competitive effects, not growth responses, favour exotic annuals. J. Appl. Ecol..

[56] Moyer S.A., Brewer J.S. (2018). Competitive responses and effects of the invasive grass *Microstegium vimineum* during oak woodland restoration. Nat. Areas J..

[57] Kraft N.J.B., Godoy O., Levine J.M. (2015). Plant functional traits and the multidimensional nature of species coexistence. Proc. Natl. Acad. Sci. U.S.A..

[58] Kleinhesselink A.R., Kraft N., Pacala S., Levine J. (2022). Detecting and interpreting higher-order interactions in ecological communities. Ecol. Lett..

[59] Hui C., Landi P., Latombe G., Traveset A, Richardson DM (2020). Plant invasions: the role of biotic interactions.

[60] Li Y., Xiao J., Liu H. (2020). Advances in higher-order interactions between organisms. Biodiv. Sci..

[61] Johnson E.C., Hastings A. (2023). Coexistence in spatiotemporally fluctuating environments. Theor. Ecol..

[62] Liu M., Rubenstein D., Cheong S. (2021). Antagonistic effects of long- and short-term environmental variation on species coexistence. Proc. Royal Soc. B.

[63] Cadotte M.W. (2023). Quantifying and linking mechanism scenarios to invasive species impact. Ecol. Appl..

[64] Altieri A.H., Irving A.D. (2017). Species coexistence and the superior ability of an invasive species to exploit a facilitation cascade habitat. PeerJ.

[65] Torres A., Morán-López T., Rodriguez-Cabal M.A. (2023). Timing of invasive species removal influences nonnative biotic resistance and trajectories of community reassembly. J. Ecol..

[66] Bowler C.H., Shoemaker L.G., Weiss-Lehman C. (2022). Positive effects of exotic species dampened by neighborhood heterogeneity. Ecology.

[67] van Kleunen M., Bossdorf O., Dawson W. (2018). The ecology and evolution of alien plants. Annu. Rev. Ecol. Evol. Syst..

[68] Allen W.J., Wainer R., Tylianakis J.M. (2020). Community-level direct and indirect impacts of an invasive plant favour exotic over native species. J. Ecol..

[69] Saavedra S., Rohr R.P., Bascompte J. (2017). A structural approach for understanding multispecies coexistence. Ecol. Monogr..

